# Effectiveness of Illness Management and Recovery program on people with severe mental illnesses: a systematic review and meta-analysis

**DOI:** 10.3389/fpsyt.2023.1162288

**Published:** 2023-05-15

**Authors:** Yong Shian Shawn Goh, Jenna Qing Yun Ow Yong, Amy Ziqiang Li

**Affiliations:** ^1^Alice Lee Centre for Nursing Studies, National University of Singapore, Singapore, Singapore; ^2^Institute of Mental Health, Singapore, Singapore

**Keywords:** Illness Management and Recovery, severe mental illness, personal-recovery, systematic review, meta-analysis

## Abstract

**Background:**

The Illness Management and Recovery (IMR) program has been established in response to the challenges faced by people with severe mental illnesses (SMIs). The program emphasizes the self-management of mental health conditions and the achievement of personally meaningful goals. However, reviews on its efficacy remain scarce, especially in recent years.

**Objective:**

This review aimed to examine the efficacy of IMR in improving personal-recovery outcomes among people with SMIs.

**Methods:**

A search was conducted on seven databases (CINAHL, Embase, ProQuest, PsycINFO, PubMed, Scopus, and Web of Science) from inception to February 2022, without limits on the dates and types of publications. Studies were included if they had examined the efficacy of IMR in one or more outcomes, investigated at least one group of participants, and been published in English. The participants were adults (at least 16 years of age) with a formal diagnosis of at least one SMI.

**Results:**

Fourteen studies were included in this review, and eight outcomes were examined: personal recovery, global functioning, social functioning, hope, perceived social support, quality of life, substance abuse, and knowledge of mental illness. There is limited evidence on the superiority of IMR to existing treatment plans or other interventions in improving the outcomes of interest among people with SMIs. However, the low attendance rates in many included studies suggest the presence of a threshold of exposure to IMR beyond which its treatment effects could be observed. Suggestions for future IMR implementation are discussed.

**Conclusions:**

The IMR program may serve as an alternative or complementary intervention for people with SMIs, especially with enhanced program exposure and access to resource materials.

**Systematic review registration:**

https://inplasy.com/inplasy-2022-10-0005/.

## Introduction

Various definitions for severe mental illnesses (SMIs) have been proposed over the years (1–3). Nonetheless, an established consensus is that the following clinical criteria have to be fulfilled in defining SMIs: (i) a diagnosis of non-organic psychosis or personality disorder; (ii) at least a two-year history of mental illness or service contact (including treatment); and (iii) disability, moderate impairment in work and non-work activities, and mild impairment in basic needs. People with SMIs experience substantial functional impediments to their ability to fully participate in society and major life activities (4). Additionally, besides barriers such as stigma (5) and employment problems (6), they face difficulties in accessing and navigating through a healthcare system (7). Collectively, such difficulties have been posited to lead to the elevated prevalence of somatic medical conditions among people with SMIs, potentially predisposing them to premature mortality (8).

Progressive decentralization of mental healthcare over recent decades might have partly been responsible for a fragmented medical system. This system not only relies on coordination between healthcare organizations, but also obliges individuals to establish and maintain contact with a multiplicity of such organizations (7, 9). The fragmentation is further confounded by existing challenges in the treatment for people with psychiatric-somatic comorbidities (10) and by difficulties faced by people with SMIs in establishing a point of contact with medical professionals (11). Against this background, people with SMIs continue to be disproportionately disadvantaged as compared with the general population.

In response to these challenges, the Illness Management and Recovery (IMR) program has been conceived to help people with SMIs acquire information and skills in managing their conditions and develop and attain personally meaningful goals (12). IMR has been developed under the National Implementing Evidence-Based Project, which focuses on the development of implementation and training materials for interventions to increase access for people with SMI (13). IMR revolves around the principles of recovery, viewing people with SMIs as individuals who can pursue meaningful goals and aspirations beyond the limitations of their conditions (14). Underpinned by the trans-theoretical and stress-vulnerability models, the IMR program, developed between 2000 and 2002, aims to improve personal and clinical recovery: it considers a given individual's stages of change and interrupts the cycle of stress and vulnerability responsible for relapses and functional impairments (15). IMR is curriculum-based and standardized, incorporating motivation-based, educational, and cognitive-behavioral strategies. As of 2011, the program comprises 11 modules: recovery strategies; facts of mental illnesses; stress-vulnerability model; social support; medication use; substance use; relapse prevention; coping with stress; coping with persistent symptoms; getting needs met in the mental healthcare system; and healthy lifestyles (16). The program had an earlier edition in 2006 (15), consisting of nine modules (excluding substance use and healthy lifestyles). IMR typically spans 6–12 months and may be conducted individually or in groups (16). Online resources are also available, such as educational handouts and practitioner guidelines on the Substance Abuse and Mental Health Services Administration (SAMHSA) website (17), thus making IMR accessible to practitioners globally.

To our knowledge, there has hitherto been one review published in 2014 on the IMR program that included studies published before June 2011 (18), a systematic review on self-management interventions which included IMR (19), and a review protocol (20) with similar outcomes as this paper. Another review published in 2002 compared 40 randomized controlled trials (RCTs) in individual components of IMR, such as knowledge of mental illness, medication adherence, symptom relapses and rates of re-hospitalization, and severity and distress of persistent symptoms (21). Additionally, McGuire, Kukla (18) explored client and implementation outcomes based on a combination of RCTs, quasi-controlled, and pre-post trials. In this context, with no reviews on this field of research for over a decade, an updated systematic review is critical as it examines the effectiveness of the IMR program in modern recovery interventions.

## Aims

This review aimed to explore the effectiveness of IMR in improving personal-recovery outcomes among people with SMIs. Our specific research question was: compared with the standard care or other interventions, how effective are IMR programs in improving personal-recovery outcomes among people with SMIs?

### Methods

To ensure methodological rigor (22), this review was guided by the Preferred Reporting Items for Systematic Reviews and Meta-Analyses (PRISMA) 2020 statement (23). In addition, a review protocol has been registered on INPLASY2022100005.

### Search strategy

To ensure a comprehensive and updated search for studies on IMR programs for people with SMIs, specific keywords and Medical Subject Headings (MeSH) terms were formulated with the help from the University librarian. These included “illness management and recovery”, “IMR”, “mental disorders” [MeSH], “mental illness”, “schizophrenia”, “bipolar”, and “psychosis”. Based on Boolean operators, seven databases were searched from inception to February 2022, with coverage across multiple disciplines (Scopus and Web of Science) and specific disciplines, including biomedicine (Embase, ProQuest, and Pubmed), nursing and allied health (CINAHL), and psychology (PsycINFO). To avoid omission of relevant materials, no limits were applied to the types and years of publications, and studies citing previous reviews (18, 19) were also retrieved. Given the lack of access to interpreters, only publications in English were included.

### Eligibility criteria

#### Population

Participants in the included studies were adults (at least 18 years of age) diagnosed with schizophrenia, schizophreniform, schizoaffective, bipolar, or mood disorders. The diagnoses were based on a psychiatrist's clinical judgement or standardized criteria such as the Diagnostic and Statistical Manual of Mental Disorders (DSM-V) or the International Classification of Diseases (ICD-10). Participants in the included studies were inpatients or individuals recruited from outpatient programs.

#### Intervention

The included studies were RCTs examining the efficacy of IMR among people with SMIs. The studies were required to have adhered to the standardized, curriculum-based IMR program based on the principles of recovery, with a focus on the following aspects: psycho-education for mental illnesses; cognitive-behavioral approaches to medication adherence; developing plans for relapse prevention; training for social skills; and skills to cope and manage symptoms (15). These five strategies may be implemented through the eleven or nine modules from the IMR manuals (15, 16) or adapted according to the population's needs.

#### Comparator

Studies with no comparators (participants receiving no interventions), a passive comparator (usual-care or wait-list control groups), or an active comparator (other interventions) were included.

#### Outcomes

The primary outcomes of this review included changes in global functioning and personal recovery for people with SMIs. Secondary outcomes included specific areas of functioning (such as social functioning), specific areas of personal recovery (such as hope and perceived social support), substance abuse, quality of life, and knowledge of the illness.

### Selection of articles

All retrieved studies from the database and end-reference list search of included studies were uploaded into EndNote X9, where duplicates were electronically removed. The titles and abstracts of the remaining studies were screened independently by two reviewers (Authors 1 and 3); those not meeting the inclusion and exclusion criteria were removed at this stage. Studies deemed potentially suitable by at least one author were then downloaded and reviewed independently by Authors 1 and 3. Any disagreements between them were resolved by means of consensus through discussions. Protocols, abstracts, and publications not in English were excluded.

### Data extraction

Study-related data (authors, locations, years, research designs, and sample sizes) and outcome measures were extracted by Authors 1 and 3. For studies with missing data on numerical outcomes, their authors were contacted for clarification. A pilot review was conducted by the two reviewers based on a data-extraction form adapted from the Cochrane Handbook for Systematic Reviews of Interventions to ensure consensus during data extraction (24). Any disagreements were resolved through discussions.

### Risk of bias

The risk of bias (ROB) of the included studies was evaluated independently by Authors 1 and 3 based on the Cochrane ROB assessment tool (24). Relevant aspects included allocation concealment, blinding of outcome assessments, blinding of personnel, incomplete outcome data, random sequence generation, and selective reporting. For each study, the domains of bias were individually rated “low risk,” “high risk,” or “unclear risk,” and any disagreements were resolved through discussions. A ROB summary graph was then generated by the Review Manager 5.4 software (25).

### Data analysis

For comparative analysis, post-program measurements were extracted from each of the studies. For each continuous outcome, the mean difference (MD) and its 95% confidence intervals (CIs) were computed as measures of treatment effects. Statistical heterogeneity between the studies was examined through the Chi-square test and *I*^2^ statistics: a statistically significant Chi-square *P* value (*P* < 0.10), accompanied by an *I*^2^ statistic of at least 50%, was interpreted as evidence of significant heterogeneity. A fixed-effect model was adopted for homogeneous studies; otherwise, a restricted maximum likelihood random-effects model was used (26). Aggregation of effect sizes was chosen over other methods for studies with the problem of effect-size multiplicity, given the limited number of studies examined in each meta-analysis in this review (27). The effect sizes in all meta-analyses were measured through Hedges' g statistic (28). All data were analyzed through the RStudio software (29).

## Results

**Figure 1:**
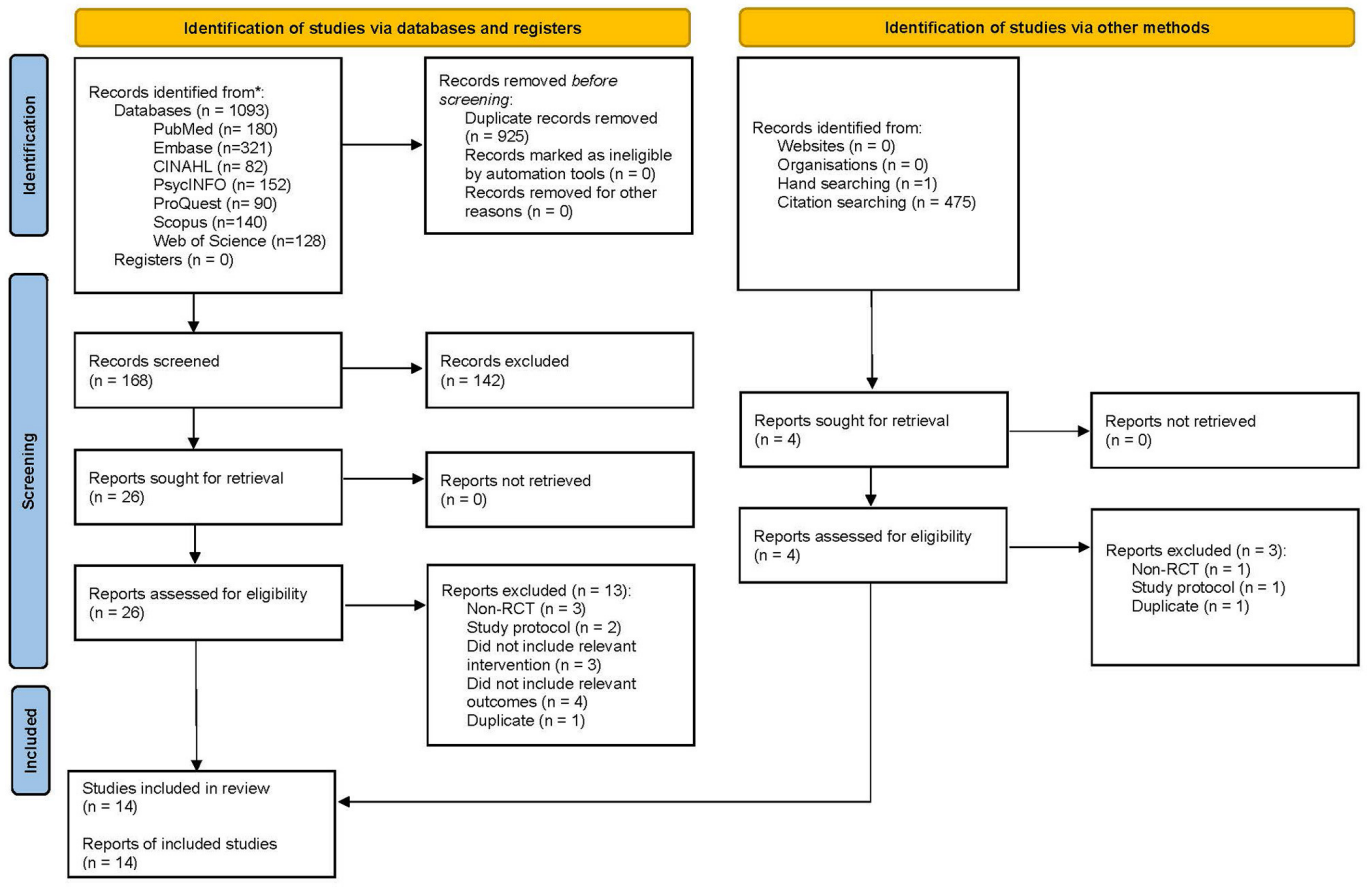
PRISMA 2020 flow diagram (23).

Publications were retrieved not only from databases but also from citations- and hand-searching. Of the 1,093 publications retrieved from the databases, 925 duplicates were removed. Upon title- and abstract screening, another 142 were removed. Full-text evaluation of the remaining 26 publications led to removing 13 (with reasons), leaving behind only 13. Additionally, of the 476 publications retrieved from citation- and hand-searching, four remained after duplicate removal and title- and abstract screening. Upon full-text evaluation, another three publications were removed (with reasons), leaving behind only one. These thus led to the final inclusion of 14 studies in this review (Figure 1).

### Characteristics of the included studies

The included studies were published between 2007 and 2021 across various countries: Turkey (30); the Netherlands (31); Singapore (32); Denmark (33–35); the United States of America (10, 36–39); Sweden (40); Taiwan (41); and Israel (42). Their sample sizes ranged from 34 (39) to 324 (38), with a median of 152 (Table 1).

**Table 1:** Selected characteristics of included studies.

**Study ID**	**References**	**Condition examined**	**Country**	**Aim of study**	**IMR program modules**	**Sample size**	**Groups**	**Measure of primary outcomes**	**Results**
0020	Dalum et al. (33)	Schizophrenia or bipolar disorder	Denmark	To investigate the benefits and harms of the Illness Management and Recovery (IMR) program among people with severe mental illness in Denmark. IMR builds among other approaches on a psychoeducational approach.	11	IMR+TAU: 99. TAU: (99)	Intervention: IMR + TAU Comparator: TAU	Global functioning Did not measure. Personal recovery 1. IMRS (Client) 2. IMRS (Clinician) MHRM	Personal recovery Between-groups: No statistical differences were found between the two groups in the intention-to-treat analyses of IMRS (Client): *t(1)* = 1.49, *p* = 0.14, and IMRS (Clinician): *t(1)* = 0.31, *p* = 0.76. No statistical differences were found between the two groups in the complete case analyses of MHRM (*p* = 0.90).
0048	Dalum et al. (34)	Schizophrenia or bipolar disorder	Denmark	To investigate the benefits and harms of the IMR program compared with treatment as usual in Danish patients with schizophrenia or bipolar disorder.	11	IMR+TAU: 99. TAU: 99	Intervention: IMR + TAU Comparator: TAU	Global functioning Global Assessment of Functioning (functioning) (GAF-F) Personal recovery Did not mention	Global functioning Between-groups: No statistical differences were found between the two groups in the intention-to-treat and complete cases analyses of GAF-F (*p* = 0.21)
0076	Färdig, et al. (40)	Schizophrenia or schizoaffective disorder	Sweden	To evaluate the effects of the illness management and recovery (IMR) program on symptoms and psychosocial functioning of individuals with schizophrenia or schizoaffective dis-order in an outpatient setting in Sweden.	9	IMR:21. TAU: 20.	Intervention: IMR Comparator: TAU	Global functioning Did not mention. Personal recovery 1. IMRS (Client) 2. IMRS (Clinician) 3.RAS	Personal recovery Changes in personal recovery: Compared with participants in treatment as usual, those in the IMR program demonstrated greater improvement in illness management as measured on the self-reported (*p* = 0.001) and clinician-reported (*p* < 0.001) versions of the IMRS. However, no statistically significant differences were observed for either of the groups between assessment points for RAS factors (*p* =0.808). Between-groups: Statistically significant differences were found between self-reported and clinician-reported IMRS ratings at both posttreatment and follow-up, when the analyses controlled for baseline ratings.
0101	Hasson-Ohayon et al. (42)	Severe mental illness	Israel	To evaluate the effectiveness of the Illness Management and Recovery program implemented in a group format.	9	IMR: 119. TAU: 91	Intervention: IMR Comparator: TAU	Global functioning Did not mention. Personal recovery 1. IMRS (Client) 2. IMRS (Clinician)	Personal recovery Changes in personal recovery: Statistically significant interactions between group and time were found for the total Illness Management and Recovery Scale completed by clinicians (F=4.18, df=1 and 146, *p < 0.0*5) There was also a trend to- ward significance between group and time for the total Illness Management and Recovery Scale completed by clients (F=3.64, df=1 and 148, *p < 0.0*6). This indicates that from the perspectives of both the client and the clinician, Illness Management and Recovery Scale total scores improved significantly more for the participants in the program than for those who received treatment as usual. Between-groups: For the clinician Illness Management and Recovery Scale total scores, both the group and time main effects were statistically significant (F=7.60, df=1 and 146, *p < 0.0*1 and F=41.16, df=1 and 146, *p < 0.0*01, respectively). This indicates that from the clinicians' perspective, participants in the intervention received higher total scores on the Illness Management and Recovery Scale before initiation of the intervention and upon its completion. Also, on the basis of the clinicians' scoring all participants, regardless of whether they were in the intervention group, received higher total scores at the completion of the intervention. From the clients' perspective, statistically significant main effects emerged for the Illness Management and Recovery Scale total score (group, F=7.20, df=1 and 148, *p < 0.0*1).
0074	Jensen et al. (35)	Schizophrenia or bipolar disorder	Denmark	To improve upon the methodological limitations of previous IMR research and to evaluate whether participants in the IMR pro-gram exhibited improved clinical and personal recovery and illness management postintervention and at the one-year follow-up	11	IMR+TAU: 99. TAU: 99	Intervention: IMR + TAU Comparator: TAU	Global functioning GAF-F Personal recovery 1. IMRS (Client) 2. IMRS (Clinician) 3. MHRM	Global functioning Between-groups: In the GAF-F, a nonsignificant group indicated a difference of 0.8 in favor of the IMR group (95% confidence inter-val [CI]: – 4.7 to 3.0 points, t = – 0.42 p = 0.45). Personal recovery Between-groups: Analyses of changes in personal recovery based on the MHRM showed no differences between the IMR and control groups between either the baseline and one-year follow-up or the postintervention and one-year follow-up time points. There were no significant differences found for the two scales of IMRS between the two groups. As a post hoc analysis, we examined the time effect and both groups improved on functioning (GAF-F) and personal recovery (MHRM) but not on IMRS (data not shown).
1032	Johnson (39)	Severe and persistent mental illness	USA	To examine how patient mental health recovery is affected by Illness Management and Recovery treatment compared to psychological treatment-as-usual at Madison State Hospital	9 (with optional module for substance use)	IMR: 19. TAU: 15	Intervention: IMR Comparator: TAU	Global functioning Measured but analyses conducted were not relevant. Personal recovery MHRM	Personal recovery Changes in personal recovery: •There was a statistically significant difference from pre-test to post-test for the IMR treatment group (*p =* 0.000). •There was no statistically significant difference from pre-test to post-test for the psychological TAU group (*p =* 0.218). Between-groups: •The results of the t-test indicated that there was a statistically significant difference in change scores by group (*p* = 0.000). Therefore, the participants in the IMR group had significantly different change scores than those in the psychological TAU group. •In conclusion, the participants in the IMR group had greater change scores than those in the psychological TAU group.
0062	Levitt et al. (37)	Serious and persistent mental illness	USA	To evaluate the effects of the illness management and recovery program on mental illness and functional outcomes of persons with serious mental illness who were receiving supportive housing services.	10 (9 modules + substance use)	IMR: 54. WL: 50	Intervention: IMR Comparator: Waiting List (WL)	Global functioning Did not mention Personal recovery 1. IMRS (Client) 2. IMRS (Clinician)	Personal recovery Changes in personal recovery: •Compared with those on the waitlist, the program participants demonstrated significantly greater gains in illness self-management as measured on both the client IMRS (*F*(1, 89) = 8.68, *p* = 0.002) and clinician IMRS (*F*(1, 93) = 12.52, *p* = 0.001).
0084	Lin et al. (41)	Schizophrenia	Taiwan, China	To evaluate the feasibility and effects of an IMR program adapted for individuals with schizophrenia who were awaiting discharge into the community.	Adapted IMR for acute care setting (based on 3 modules: practical facts about Schizophrenia, using medication effectively, and coping with problems and persistent symptoms)	IMR: 48. TAU: 49	Intervention: IMR Comparator: TAU	Global functioning Did not mention. Personal recovery Did not mention.	N.A.
0224	Muralidharan et al. (10)	Serious mental illness	USA	To examine Living Well, a group-based illness self-management intervention for adults with serious mental illness that was cofacilitated by two providers, one of whom has lived experience with co-occurring mental health and medical conditions.	Did not mention	Intervention: 124. Control: 118	Intervention: Living Well intervention Comparator: Medical Illness Education and Support group (active control group)	Global functioning Did not mention. Personal recovery Maryland Assessment of Recovery Scale (MARS)	Personal recovery Changes in personal recovery: •There was a nonsignificant trend for greater improvement on the MARS among Living Well participants at posttreatment but not at follow-up.
0003	Polat and Kutlu (30)	Schizophrenia	Turkey	To determine the effect of the illness management and recovery program in patients with schizophrenia	10 (9 modules + substance use)	IMR: 25. TAU: 25	Intervention; Illness Management and Recovery program (IMR) Comparator: Treatment-as-usual (TAU)	Global functioning Did not measure. Personal recovery 1. Illness Management and Recovery Scale (IMRS) (Patient) 2. IMRS (Clinician)	Personal recovery Changes in personal recovery: A statistically significant difference in IMRS-P scores was observed within the intervention group for pre-test, post-test, and follow-up points, *Friedman test = 34.86, p < 0.0*01. A statistically significant difference in IMRS-P scores was observed within the control group for pre-test, post-test, and follow-up points, *Friedman test = 22.80, p < 0.0*01. Between-groups: *Post-test* A statistically significant difference in IMRS-P scores was observed between groups, *t = 4.928, p < 0.0*01. *1-month follow-up:* A statistically significant difference in IMRS-P scores was observed between groups, *t = 5.863, p < 0.0*01.
0008	Roosenschoon et al. (31)	Schizophrenia or a persistent mood disorder with or without comorbid disorders	The Netherlands	To comprehensively investigate the effectiveness of IMR, including the impact of completion and fidelity	11	IMR+TAU: 116. TAU: 71	Intervention; IMR + TAU Comparator: TAU	Global functioning Did not measure. Personal recovery 1. IMRS (Client) 2. IMRS (Clinician) 3. Mental Health Recovery Measure (MHRM)	Personal recovery Changes in personal recovery: As compared with the control group, the IMR group showed a statistically significant improvement in the client version of the IMR scale (p = 0.048). As compared with the control group, the IMR group did not have statistically significant improvement in the clinician version of the IMR scale (p = 0.180). As compared with the control group, the IMR group did not have statistically significant improvement in the MHRM (p = 0.100). Both the experimental and control group showed statistically significant improvement over time, as measured using the clinician version of the IMR scale (*p* = 0.007).
118	Salyers et al. (38)	Schizophrenia, bipolar disorder, or other major mood disorders	USA	To examine the integration of two evidence-based practices for adults with severe mental illness: Assertive community treatment (ACT) and illness management and recovery (IMR) with peer specialists as IMR practitioners.	10 (9 modules + substance use)	IMR+ACT: 183. ACT: 141	Intervention: IMR + ACT Comparator: ACT	Global functioning Did not mention. Personal recovery 1. IMRS (Client) 2. IMRS (Clinician)	Personal recovery Changes in personal recovery: There was one significant time effect, with clinician ratings of client illness self-management showing improvements over time, F(2, 188) = 7.42, *p* < 0.001. However, this did not differ by condition. Between-groups: We examined the hypothesis that consumers in ACT–IMR programs would have greater improvements in illness self-management over time. However, the hypotheses were not supported. Consumers across the two conditions did not improve on these measures over time and the conditions did not differ in general, or in their rate of change over time.
0051	Salyers et al. (36)	Schizophrenia or schizoaffective disorder	USA	To rigorously test Illness Management and Recovery (IMR) against an active control group in a sample that included veterans.	10 (9 modules + substance use)	Intervention: 60. Control: 58	Intervention: IMR + TAU Comparator: Intensive problem-solving + TAU	Global functioning Did not mention Personal recovery 1. IMRS (Client) Recovery Assessment Scale (RAS)	Personal recovery Changes in personal recovery: IMRS (Client): Participants in both groups improved significantly across time periods, *F*(2, 80) = 3.55, *p* = 0.05. RAS: No significant improvements were found (*p* >0.05). Between-groups: •Analyses of mean-response profiles revealed no group differences between the two groups on IMRS or RAS (*p>*0.05).
0016	Tan et al. (32)	Schizophrenia, bipolar, depression, anxiety disorder or schizoaffective disorder	Singapore	To To evaluate the effectiveness of the Illness Management and Recovery Program in comparison with the current standard of care in terms of reduction of symptoms, rehospitalisation rates and social functioning in Asia	10 (9 modules + substance use)	IMR: 25. TAU: 25	Intervention: IMR Comparator: TAU	Global functioning Global Assessment Scale (GAS) Personal recovery •IMRS (Client) •IMRS (Clinician	Global functioning Changes in global functioning:
									Results indicated that the interaction between time and group (F (2.50, 105.03) = 69.13, p = 0.000, η2p = 0.62) was also significant in predicting GAS. Participants in the intervention group were found to gain significant improvement gradually from baseline to 6 months, 12 months, as well as 24 months whereas participants in control group were reported to deteriorate from baseline to 6 months later and continue not to improve in both 12 months and 24 months later. Between-groups: There was an overall statistically significant difference between intervention and control groups (F (1, 42) = 84.27, p = 0.000, η2p = 0.67). Personal recovery Changes in personal recovery: IMRS (client): The interaction between time and group (F (2.42, 101.58) = 167.08, p = 0.000, η2p = 0.80) was also found to be significant. Participants in the intervention group generally agreed that they improved within the first 6 months from base-line and this improvement lasted even after 12 months and 24 months. On the other hand, participants in the control group did not report this improvement.•IMRS (clinician): Results showed that interaction between time and group (F (2.56, 107.40) = 145.96, p = 0.000, η2p = 0.78) was significant. In the simple effect analyses, we noticed the clinician's ratings improved significantly as early as 6 months later as compared to baseline, this improvement remained 12 months and 24 months later. Nevertheless, the ratings for, 6 months, 12 months and 24 months fluctuated in the control group. Between-groups:
									IMRS (client): There was an overall statistically significant difference between intervention and control groups (F (1, 42) = 375.23, p = 0.000, η2p = 0.90). •IMRS (clinician): There was an overall statistically significant difference between intervention and control groups (F (1, 42) = 413.57, p = 0.000, η2p = 0.91).

Of the 14 studies, six compared the IMR intervention group with a treatment-as-usual (TAU) group (30, 32, 39–42). Another four studies compared a group receiving IMR and TAU with another group receiving only TAU (31, 33–35). One study compared a group receiving IMR and TAU with a wait-list (WL) group (37). Lastly, three studies compared a group receiving IMR with another group receiving only other interventions (10, 36, 38).

### Risk of bias in the studies

**Figure 2:**
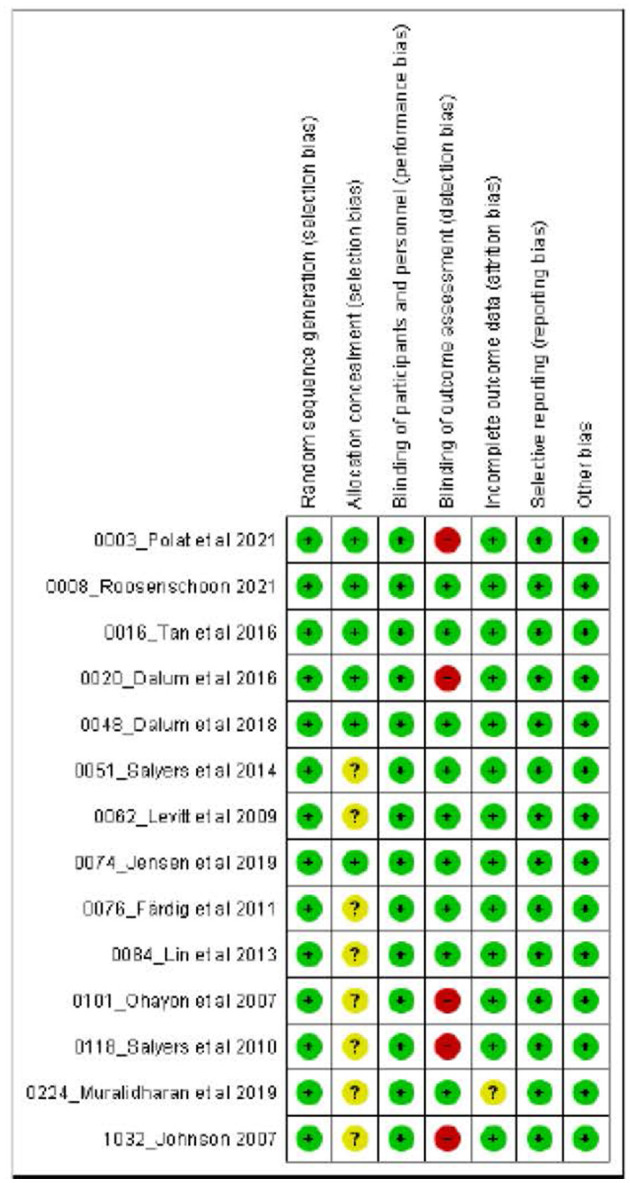
Risk of bias summary.

Most of the 14 included studies (Figure 2) were assessed to have a low overall ROB. Over half of them registered an unclear risk originating chiefly from allocation concealment, on which insufficient information had been provided in the studies. Several studies also registered a high risk of bias from outcome blinding due to the use of participant-reported outcomes.

### Primary outcomes personal recovery

A meta-analysis was conducted on 11 studies (10, 30–33, 35–40) for post-test complete-cases (CC) analysis and eight studies (10, 30, 31, 35–38, 40) for follow-up CC analysis. One study (42) could not be included in the post-test CC meta-analysis due to missing data. Intention-to-treat (ITT) analyses were also conducted for two studies (31, 33) for post-test data and another two studies (31, 35) for follow-ups.

**Figure 3:**
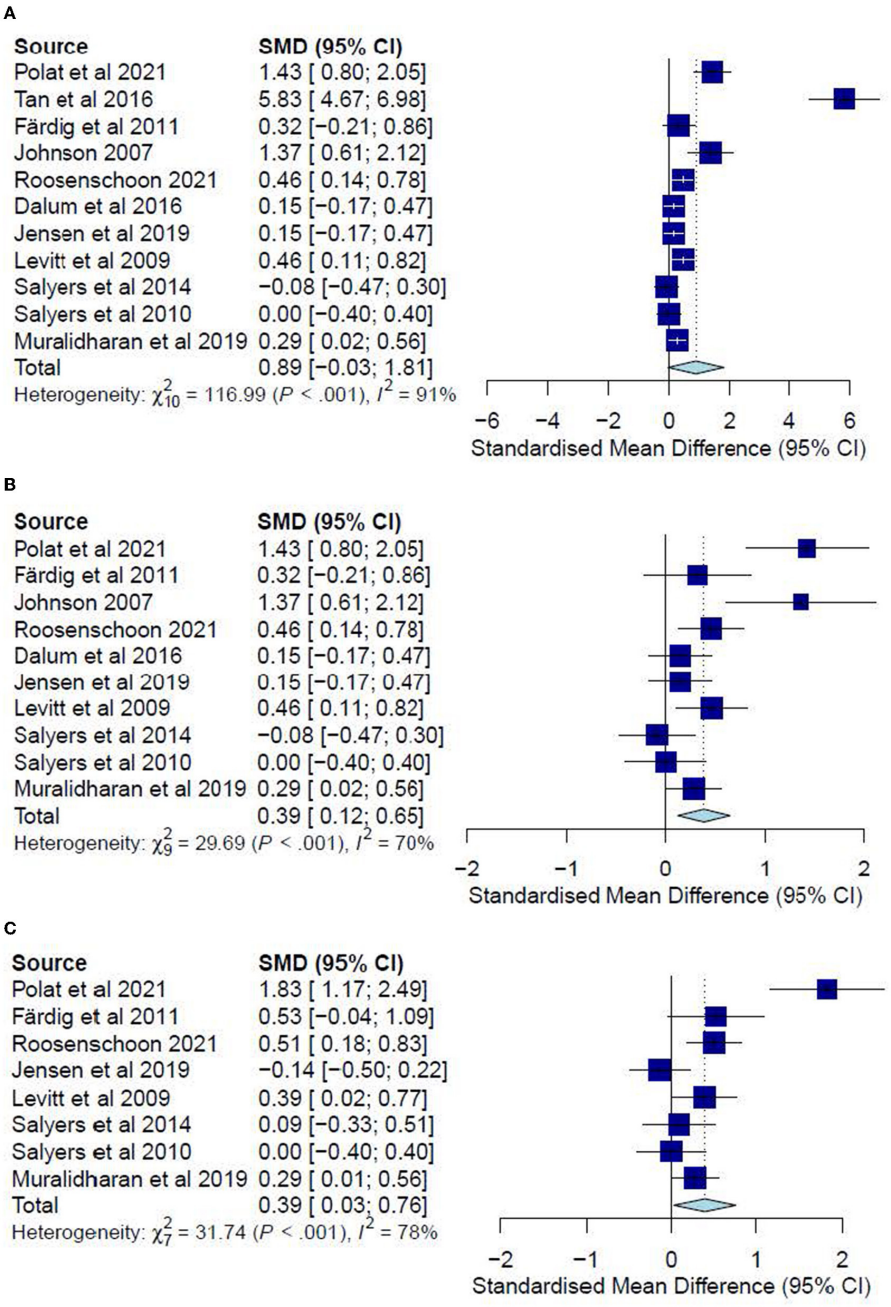
**(A)** Effectiveness of IMR program on personal recovery (post-test CC). **(B)** Effectiveness of IMR program on personal recovery (post-test CC)—after removal. **(C)** Effectiveness of IMR program on personal recovery (follow-up CC).

**Figure 4:**
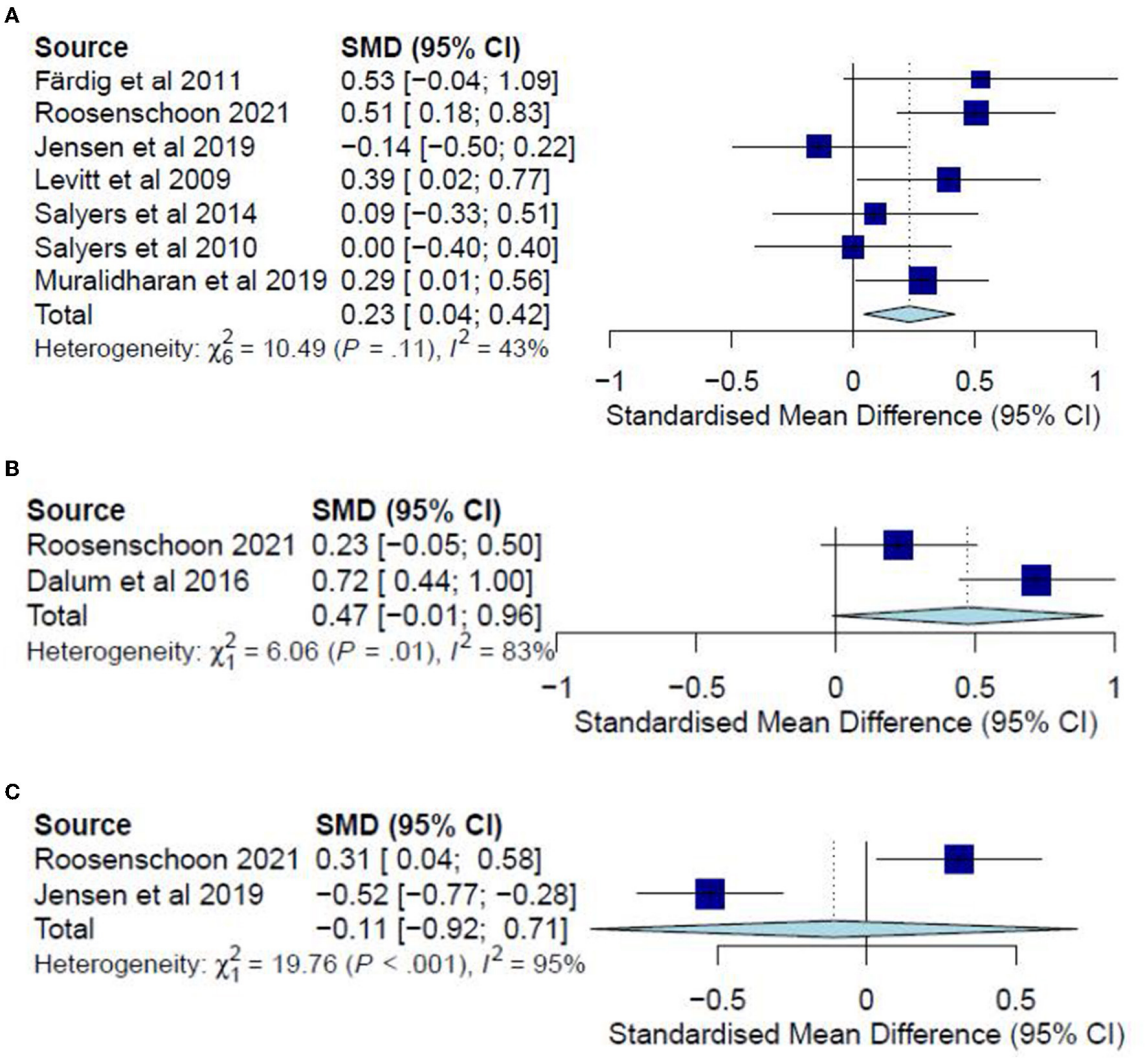
**(A)** Effectiveness of IMR program on personal recovery (follow-up CC)—after removal. **(B)** Effectiveness of IMR program on personal recovery (post-test ITT). **(C)** Effectiveness of IMR program on personal recovery (follow-up ITT).

The post-test CC meta-analysis included 1,016 participants and registered a combined standardized mean difference (SMD) of 0.89 (95% CI −0.03 to 1.81), with a heterogeneity of 91% (*P* < 0.10) (Figure 3A). A leave-one-out sensitivity analysis that excluded Tan, Ishak (32) yielded a statistically significant combined SMD of 0.39 (95% CI 0.12 to 0.65), with the heterogeneity diminishing to 70% (*P* < 0.10) (Figure 3B). Additionally, the follow-up CC meta-analysis included 743 participants and registered a combined SMD of 0.39 (95% CI 0.03 to 0.76), with a heterogeneity of 78% (*P* < 0.10) (Figure 3C). Similarly, a leave-one-out sensitivity analysis that excluded Polat and Kutlu (30) yielded a statistically significant combined SMD of 0.23 (95% CI 0.04 to 0.42), with heterogeneity diminishing to 43% (*P* > 0.10) (Figure 4A). Further subgroup analysis revealed no significant differences between studies with different comparators (*P* > 0.05) for both post-test and follow-up CC meta-analyses.

The post-test ITT analysis included 358 participants and registered a non-significant combined SMD of 0.47 (95% CI−0.01 to 0.96), with a heterogeneity of 83% (*P* < 0.10) (Figure 4B). The follow-up ITT analysis included 360 participants and registered a combined SMD of−0.11 (95% CI−0.92 to 0.71), with a heterogeneity of 95% (*P* < 0.10) (Figure 4C). Accordingly, such results suggest a modest improvement in personal-recovery scores among participants who completed the program in the IMR group during the post-program periods and follow-ups compared to those in the non-IMR groups. However, the caveat is that these results should be interpreted judiciously since no significant differences were found in the more conservative ITT analyses.

### Global functioning

A meta-analysis was conducted on three studies (32, 34, 35) for post-test CC analysis and two studies (32, 35) for follow-up CC analysis. However, no meta-analyses were conducted for post-test and follow-up ITT analyses since there was only one study for each.

**Figure 5:**
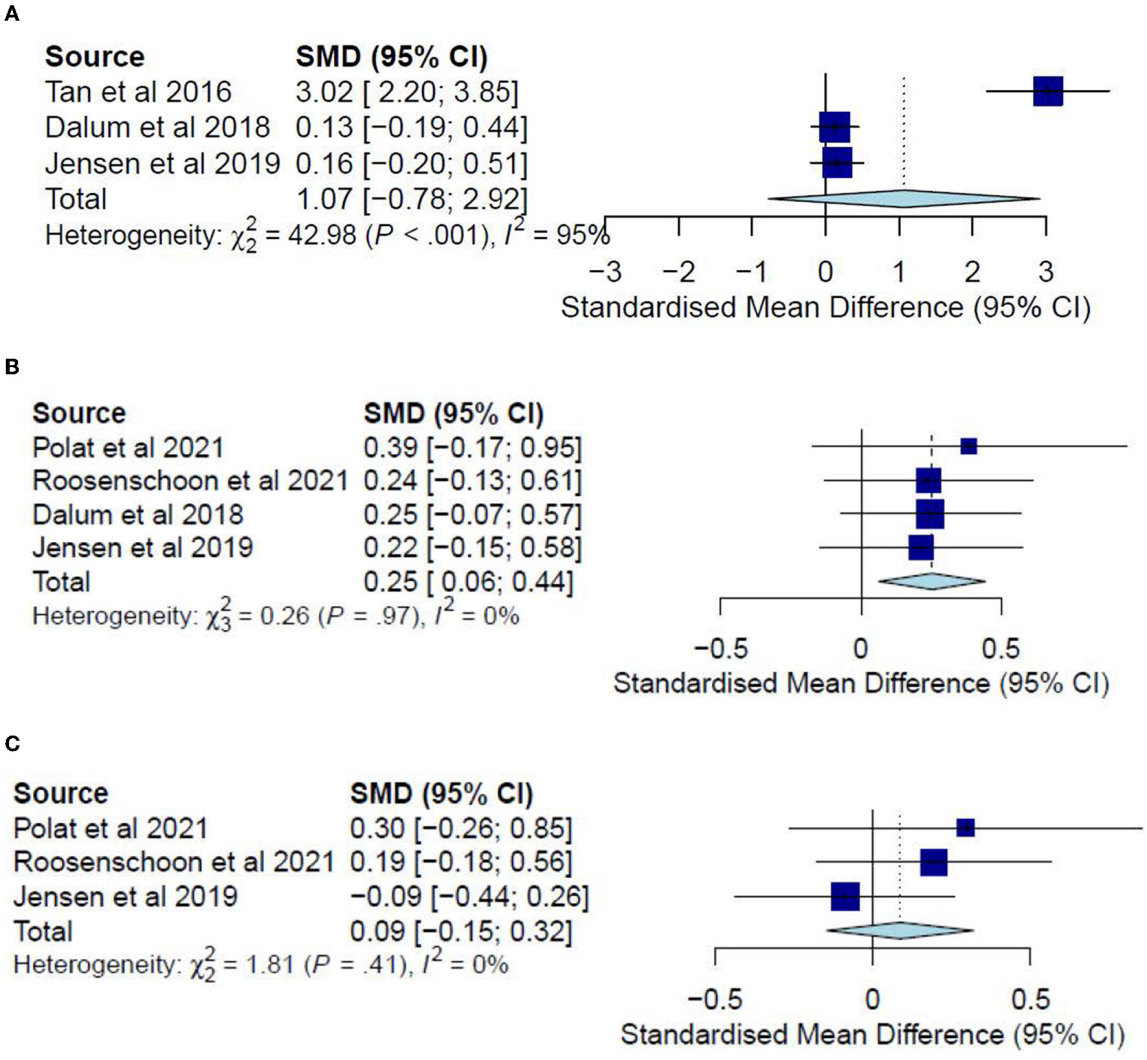
**(A)** Effectiveness of IMR program on global functioning (post-test CC). **(B)** Effectiveness of IMR program on social functioning (post-test CC). **(C)** Effectiveness of IMR program on global functioning (follow-up CC).

The post-test CC meta-analysis included 324 participants and registered a combined SMD of 1.07 (95% CI −0.78 to 2.92), with a heterogeneity of 95% (*P* < 0.001) (Figure 5A). A leave-one-out sensitivity analysis that excluded Tan, Ishak (32) yielded a non-significant combined SMD of 0.14 (95% CI −0.10 to 0.38) and heterogeneity of 0%. However, no follow-up CC meta-analysis could be conducted since the results could not be pooled due to a high heterogeneity (*I*^2^ = 98%, *P* < 0.001) arising possibly from differences in sample sizes or in measures for global functioning. In this regard, Tan, Ishak (32) included 50 participants and adopted the Global Assessment Scale, reporting significant improvements (*P* < 0.001) for the IMR group (*M* = 81.12) as compared with the TAU group (*M* = 52.2). Conversely, Jensen, Dalum (35) included 128 participants and adopted the Global Assessment of Functioning, reporting no significant differences (*P* = 0.63) between the treatment group (*M* = 50.4) and the TAU group (*M* = 50.2).

Only one study (34) measured global functioning in a post-test ITT analysis and reported non-significant differences (*P* = 0.21) between the treatment group (*M* = 46.4) and the TAU group (*M* = 44.0). Likewise, only one study (35) measured it in a follow-up ITT analysis and reported non-significant differences (*P* = 0.67) between the treatment group (*M* = 50.6) and the TAU group (*M* = 49.8). Accordingly, such results suggest only limited effectiveness of the IMR program in improving global-functioning outcomes compared with usual care.

### Secondary outcomes

#### Social functioning

A meta-analysis was conducted on four studies (30, 31, 34, 35) for post-test CC analysis and three studies (30, 31, 35) for follow-up CC analysis. In addition, ITT analyses were also conducted for two post-test studies (31, 34) and follow-ups (31, 35).

The post-test CC meta-analysis included 429 participants and registered a statistically significant combined SMD of 0.25 (95% CI 0.06 to 0.44), with a heterogeneity of 0% (*P* > 0.10) (Figure 5B). No significant subgroup differences were found (*P* = 0.62). This effect was not observed for the follow-up CC meta-analysis, which included 289 participants and registered a non-significant combined SMD of 0.09 (95% CI −0.15 to 0.32), with a heterogeneity of 0% (*P* > 0.10) (Figure 5C).

**Figure 6:**
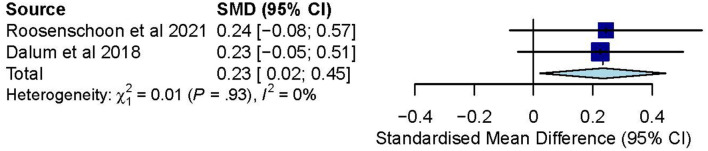
Effectiveness of IMR program on social functioning (post-test ITT).

The post-test ITT meta-analysis included 355 participants and registered a statistically significant combined SMD of 0.23 (95% CI 0.02 to 0.45), with a heterogeneity of 0% (*P* > 0.10) (Figure 6). However, no follow-up ITT meta-analysis could not be conducted due to a high heterogeneity (I^2^ = 94%, *P* < 0.10) possibly arising from differences in social functioning measurements. In this regard, Roosenschoon, van Weeghel (31) used the Social Functioning Scale and reported a significant MD of 1.90 (95% CI −0.85 to 4.65). In contrast, Jensen, Dalum (35) used the Personal and Social Performance Scale and reported non-significant differences (*P* = 0.63) between the IMR group (*M* = 52.1) and the TAU group (*M* = 53.1). Thus, such results collectively suggest that the IMR program might have been modestly better at improving social functioning than usual care, though these positive effects might not be sustained.

#### Hope

**Figure 7:**
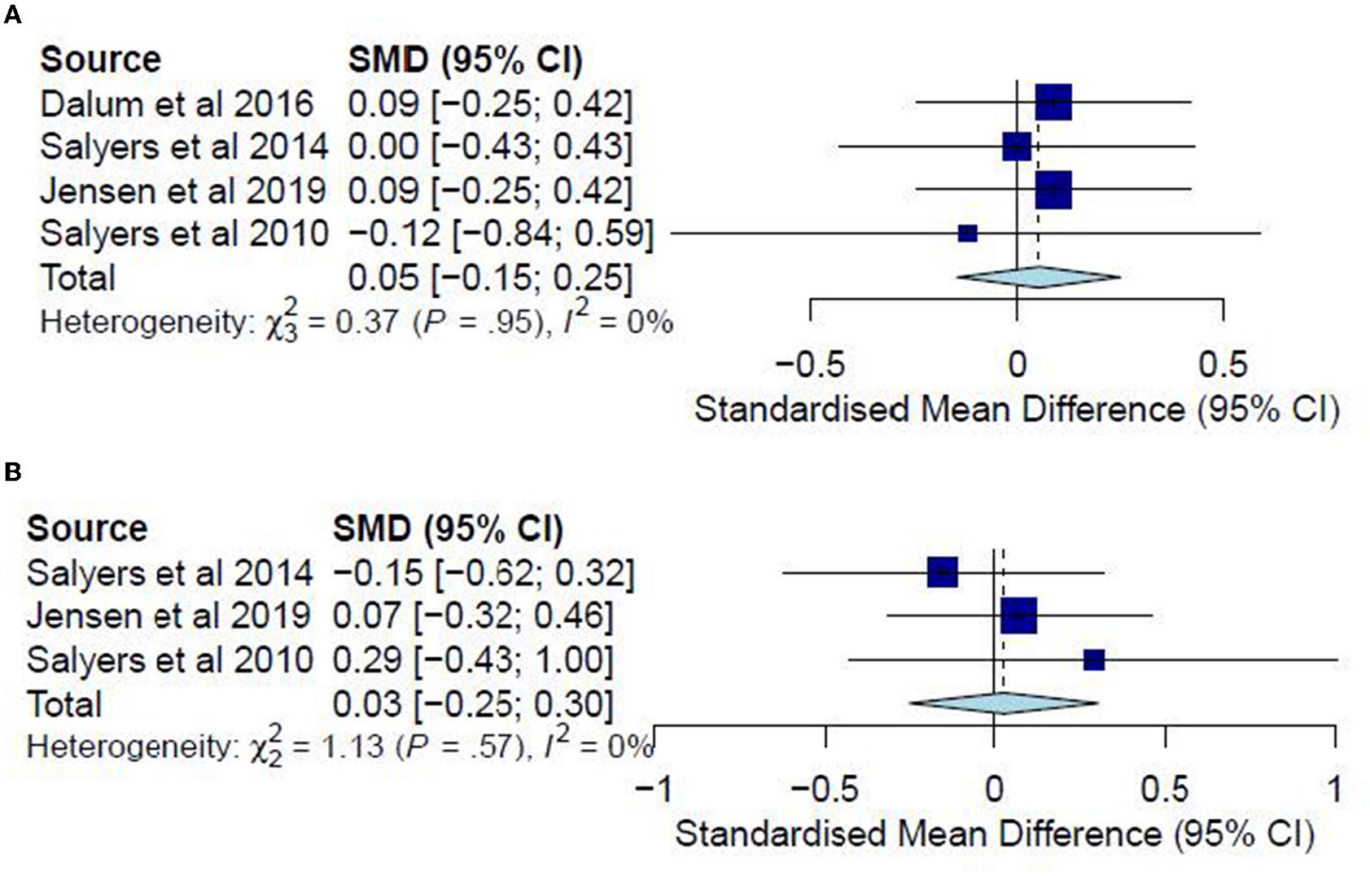
**(A)** Effectiveness of IMR program on hope (post-test CC). **(B)** Effectiveness of IMR program on hope (follow-up CC).

A meta-analysis was conducted on four studies (33, 35, 36, 38) for post-test CC analysis and three studies (35, 36, 38) for follow-up CC analysis. No meta-analyses were conducted for ITT analyses since no studies examined hope in post-test ITT analysis, and only one study did so in the follow-up analysis. The post-test CC meta-analysis included 403 participants and registered a non-significant combined SMD of 0.05 (95% CI −0.15 to 0.25), with a heterogeneity of 0% (*P* > 0.10) (Figure 7A). The follow-up CC meta-analysis included 213 participants and registered a non-significant combined SMD of 0.03 (95% CI −0.25 to 0.30), with a heterogeneity of 0% (*P* > 0.10) (Figure 7B). In addition, Jensen, Dalum (35) examined hope in a follow-up ITT analysis, reporting non-significant differences (*P* = 0.62) between the treatment group (*M* = 34.1) and the TAU group (*M* = 34.9). Accordingly, such results suggest that the IMR program has not significantly differed from usual care or other interventions in increasing hope among the participants.

#### Perceived social support

Of the two studies examining perceived social support in a post-test CC analysis, one could not be included in the post-test CC meta-analysis due to missing data (42). Hence, no meta-analysis could be conducted for this outcome. Roosenschoon, van Weeghel (31) reported a significant MD for the post-test CC analysis (0.56, 95% CI 0.08 to 1.05) but found non-significant MDs for the follow-up CC (0.43, 95% CI −0.05 to 0.91), post-test ITT (0.41, 95% CI −0.05 to 0.88), and follow-up ITT analyses (0.28, 95% CI −0.16 to 0.72).

### Quality of life

**Figure 8:**
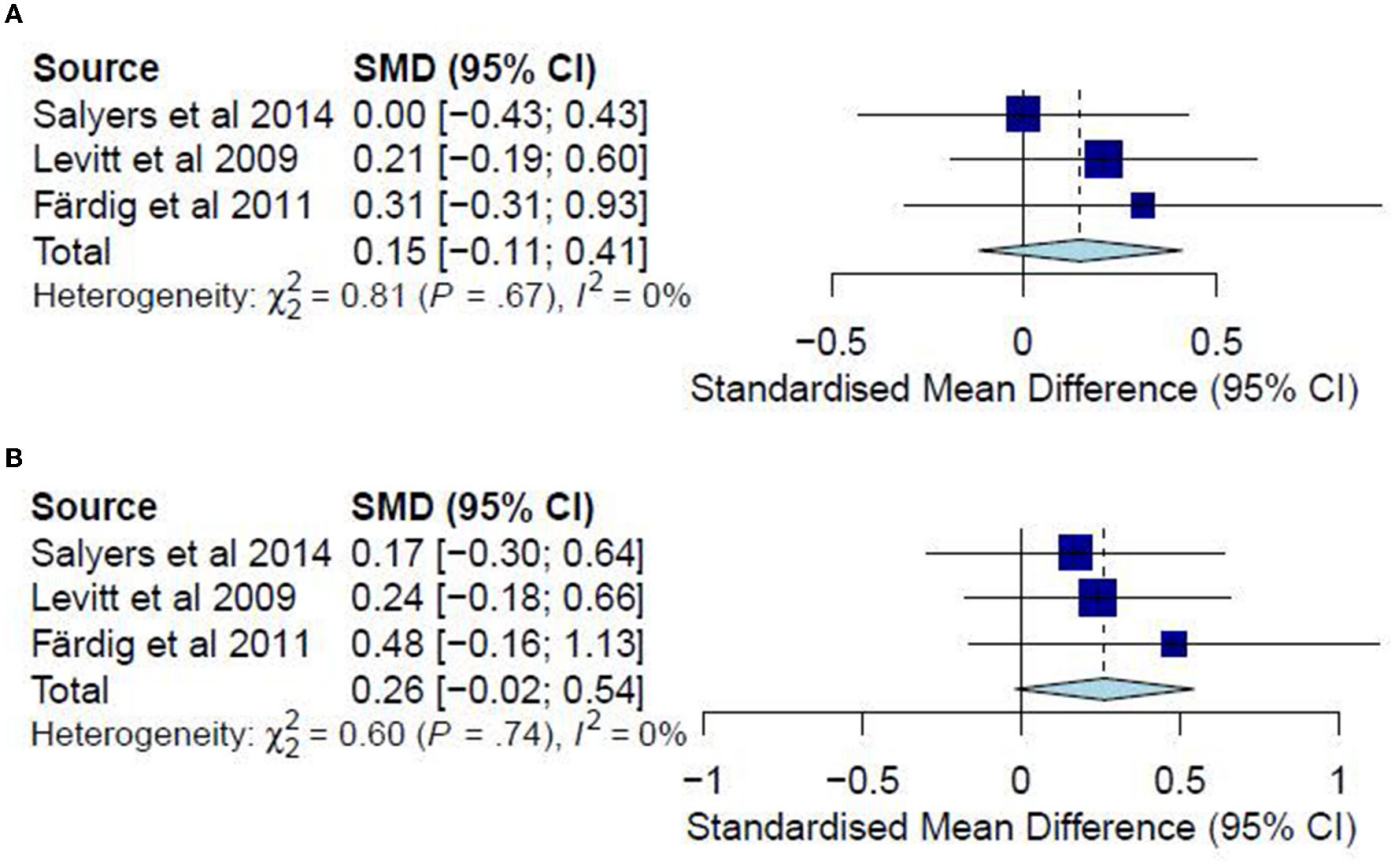
**(A)** Effectiveness of IMR program on quality of life (post-test CC). **(B)** Effectiveness of IMR program on quality of life (follow-up CC).

A meta-analysis was conducted on three studies (36, 37, 40) for post-test and follow-up CC analyses. No ITT meta-analyses were conducted since none of the included studies examined the quality of life in an ITT analysis. The post-test CC meta-analysis included 224 participants and registered a non-significant combined SMD of 0.15 (95% CI −0.11 to 0.41), with a heterogeneity of 0% (*P* > 0.10) (Figure 8A). Likewise, the follow-up CC meta-analysis included 196 participants and registered a non-significant combined SMD of 0.26 (95% CI −0.02 to 0.54), with a heterogeneity of 0% (*P* > 0.10) (Figure 8B). Accordingly, such results indicate that the IMR program has been similar to usual care, WL, and other interventions in improving the quality of life among the participants.

### Substance abuse

**Figure 9:**
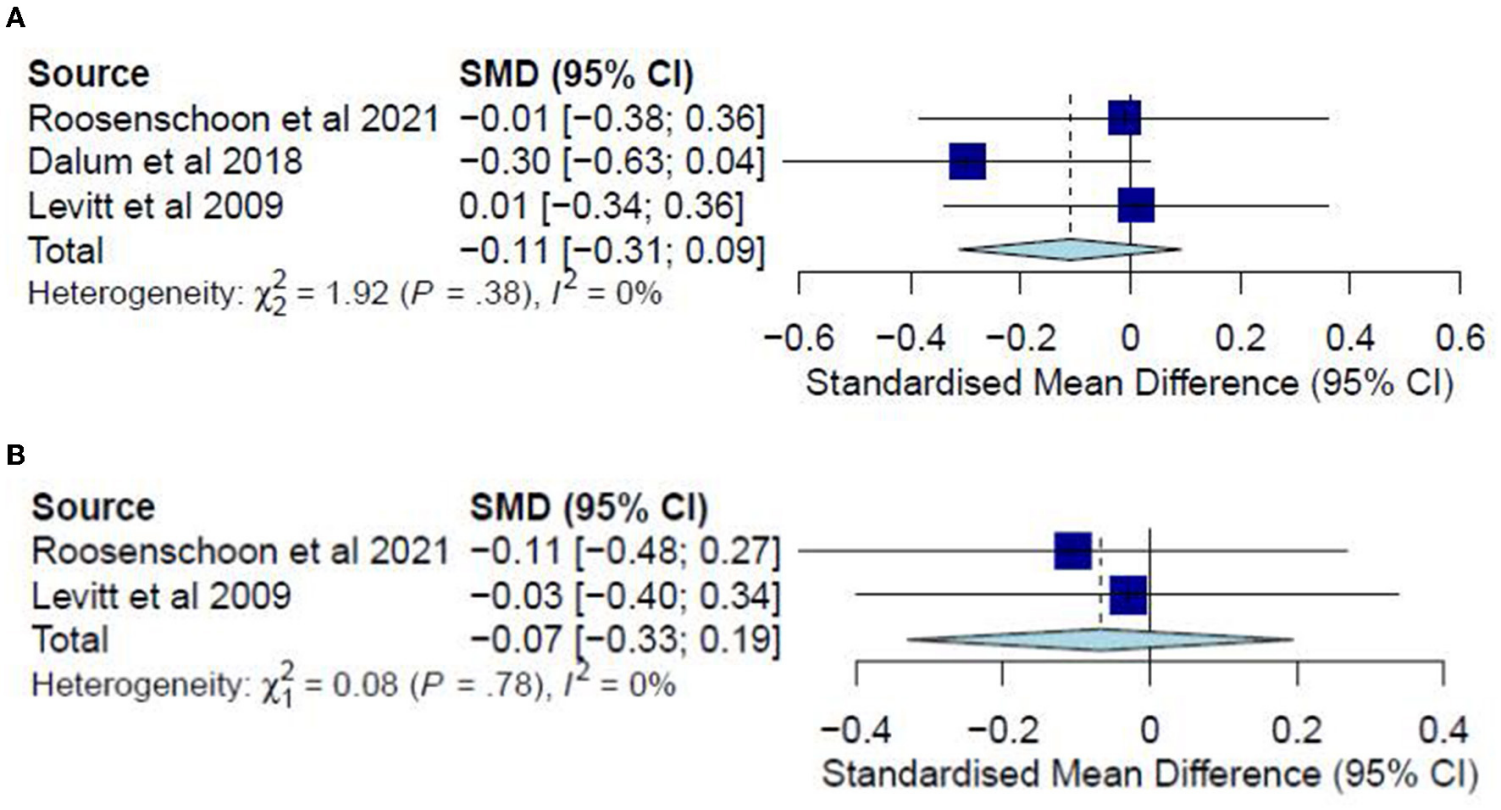
**(A)** Effectiveness of IMR program on substance abuse (post-test CC). **(B)** Effectiveness of IMR program on substance abuse (follow-up CC).

A meta-analysis was conducted on three studies (31, 34, 37) for post-test CC analysis and two studies (31, 37) for follow-up CC analysis. No ITT meta-analyses were conducted since only one study examined substance abuse in an ITT analysis. The post-test CC analysis included 352 participants and registered a non-significant combined SMD of −0.11 (95% CI −0.31 to 0.09), with a heterogeneity of 0% (*P* > 0.10) (Figure 9A). Similarly, the follow-up CC analysis included 202 participants and registered a non-significant combined SMD of −0.07 (95% CI −0.33 to 0.19), with a heterogeneity of 0% (*P* > 0.10) (Figure 9B). Furthermore, in examining substance abuse, Roosenschoon, van Weeghel (31) reported non-significant MDs between the IMR and TAU groups for both post-test (−0.09, 95% CI −0.34 to 0.17) and follow-up analyses (−0.03, 95% CI −0.38 to 0.31). Thus, such results collectively indicate that the IMR program has not significantly reduced substance abuse among the participants compared to usual care or WL conditions.

### Knowledge of mental illness

Only one study examined knowledge of mental illness (41) and reported statistically significant improvements (*P* = 0.003) for both the post-test CC analysis for the IMR group (*M* = 51.6) compared with the TAU group (*M* = 49.2). Similar results were reported in the follow-up CC analysis for the IMR group (*M* = 54.5) and TAU group (*M* = 47.1).

## Discussion

This review has examined the effectiveness of IMR programs in improving health-related outcomes among people with SMIs. Fourteen studies comparing IMR programs with passive and active comparators were included in the systematic review. However, one study (42) could not be included in any meta-analyses because of missing data. For the outcome of personal recovery among people with SMIs, the small to medium effect sizes observed in the post-program periods and at follow-ups suggest a modest advantage of IMR over TAU, WL, and other interventions. This is consistent with findings from a literature review (18).

Additionally, the absence of substantial subgroup differences in this review implied that IMR could improve personal recovery compared to active and passive intervention groups. The caveat, however, is that this finding should be judiciously interpreted since the more conservative ITT analyses have found no significant differences. For the other seven outcomes (global functioning, social functioning, hope, perceived social support, quality of life, substance abuse, and knowledge of the mental illness), IMR appeared to confer an advantage over TAU, WL, and other interventions in improving only social functioning. No benefits were discerned for the other six outcomes.

Participants' exposure to IMR (reflected by their program attendance or completion) represented a recurring determinant across the studies, of which only four achieved close to full completion rates (30, 32, 39, 40). Notably, in these four studies, the participants in the IMR group registered substantial improvements in personal-recovery scores. This finding thus suggests the presence of a threshold of exposure to IMR, beyond which its treatment effects are observable (31, 36). Moreover, further supporting evidence came from another finding in this review: improvements in personal recovery were found among those participants who had completed the IMR program in post-test and follow-up analyses but not in the ITT analyses. Furthermore, given the substantial non-adherence in the included studies, the conservative ITT analysis may have undervalued the treatment effects of IMR for those participants who were sufficiently exposed to it (43). Likewise, the absence of significant improvements in the secondary outcomes examined in this review may be due to the participants' partial exposure to the program. Nonetheless, the possibility remains that the effectiveness of IMR is not superior to other interventions or TAU. Hence, practical considerations such as the costs of implementing the program and its training should be weighed against those of other interventions.

Despite that, numerous insights for future IMR implementation emerge from this review. Firstly, while high attendance rates among people with SMIs for IMR programs may not be feasible in all intervention centers, research may be conducted to improve retention rates. This may include post-intervention interviews for participants to explore reasons for dropout and feedback on the program experience. For instance, in their qualitative interviews with subjects with low participation rates, Levitt, Mueser (37) found that those with better pre-existing knowledge of mental illness tended to drop out because of their perceived lack of benefits from the curriculum. Accordingly, pre-emptive steps should be taken to mitigate dropouts and enhance program outreach, such as stratifying participants based on their baseline knowledge (37) and tailoring the curriculum to their specific needs.

Secondly, given the severity of SMIs, some participants may require a lengthened time to complete the IMR curriculum (18), while others may tend to withdraw from the program (44). Thus, to augment the participants' exposure to the program, flexibility should be exercised, such as providing one-to-one remedial sessions for those who have missed classes or require extra assistance in specific modules in the curriculum. Additionally, such flexibility should extend to the class size of the IMR program. While individualized attention may lessen attrition among participants (44), it remains uncertain whether group sessions foster peer support and accountability that promote group cohesion and attendance (31). Most studies have adopted group sessions in this review, while only one has employed individualized home visits (32). Accordingly, future studies may compare the benefits of different IMR modalities (group-based, individualized, or mixed-model) on people with SMIs.

Lastly, the involvement of family, significant others (33, 35), and other treatment providers (36) appeared to be lacking in the IMR programs. However, evidence has demonstrated the importance of social networks in supporting the recovery of people with SMIs (45), as aided predominantly by their family members and care professionals (46). Therefore, it is unsurprising that many people with SMIs wish for greater family involvement in their care (47) and that such social support networks may mitigate the dropout rates from treatment (44). Against this background, it has been recommended that the IMR curriculum be better communicated to family members of people with SMIs and that the program be integrated with other services they use. For instance, family members can be invited to attend IMR sessions together. In addition, meetings may be held with the person with SMI, their family member, and other treatment providers to discuss and align recovery goals (48). Such recommendations may reinforce the skills and knowledge learned from IMR and enhance program attendance (36). In addition, the involvement of family and significant others would provide an important social network supporting the recovery of people with SMIs. However, the involvement of family members for people with SMI in such community interventions is often complex and involves multiple barriers (49) and may warrant the need for further research.

Aside from the possible implementation issues discussed, the effectiveness of the IMR program may have been affected by the content delivered within the modules. Given that the latest edition of the program was published in 2011, a possibility exists that the content covered may no longer be relevant in the modern context or that additional content may be required. Hence, an updated review of the program content may be timely to address the current needs of people with SMIs.

### Strengths and limitations

This review contributes to the literature on the recovery of people with SMIs by providing an updated examination of the effectiveness of the IMR program in improving various outcomes among them. However, some limitations are noteworthy, one of which is the possible omission of relevant studies, given the inclusion of only publications in English. Others include the substantial heterogeneity in some analyses, the lack of studies examining specific outcomes such as knowledge of mental illnesses, and the limited number of studies comparing IMR with other active interventions. Overall, such shortcomings may limit the conclusions that could be drawn.

## Conclusions

The IMR program incorporates motivation-based, educational, and cognitive-behavioral strategies encompassing multiple modules to enhance recovery among people with SMIs. However, the limited evidence presented in this review suggests that the program may not be significantly superior to existing treatment plans or other interventions. Nonetheless, the small to medium treatment effects observed in this paper suggest that the IMR program may benefit from further review and research, especially with regard to the implementation of the program.

### Relevance to clinical practice

Although this review did not show a superiority of the illness management and recovery program over existing treatment programs, small to medium effect sizes were observed on all personal–recovery outcomes in the post-program periods and at follow-ups, suggesting it has an advantage over other interventions. As suggested by this review, the exposure to the IMR program showed a difference between treatment effects among the participants. Therefore, mental health nurses should note this and consider providing the IMR program on a platform that can have better exposure. With the advancement of technologies, the program can be provided online or within a mobile application, which can help ensure a more sustainable exposure threshold. Having the program over such a delivery modality would give autonomy and flexibility to the participants when self-managing their recovery journey. Finally, mental health nurses should consider involving family members and significant others in the IMR program when the client starts their participation. This would help reinforce the skills and knowledge the client learned from IMR and enhance their program attendance.

## Data availability statement

The original contributions presented in the study are included in the article/supplementary material, further inquiries can be directed to the corresponding author.

## Author contributions

All authors listed have made a substantial, direct, and intellectual contribution to the work and approved it for publication.
